# Enforced PGC-1α expression promotes CD8 T cell fitness, memory formation and antitumor immunity

**DOI:** 10.1038/s41423-020-0365-3

**Published:** 2020-02-13

**Authors:** Nina Dumauthioz, Benjamin Tschumi, Mathias Wenes, Bastien Marti, Haiping Wang, Fabien Franco, Wenhui Li, Isabel C. Lopez-Mejia, Lluis Fajas, Ping-Chih Ho, Alena Donda, Pedro Romero, Lianjun Zhang

**Affiliations:** 1 Translational Tumor Immunology Group, Ludwig Cancer Research, University of Lausanne, Epalinges, Switzerland; 2 Department of Fundamental Oncology, University of Lausanne, Epalinges, Switzerland; 3 Ludwig Cancer Research Institute Lausanne Branch, Epalinges, Switzerland; 4 Center for Systems Medicine, Institute of Basic Medical Sciences, Chinese Academy of Medical Sciences and Peking Union Medical College, 100005 Beijing, China; 5 Suzhou Institute of Systems Medicine, Suzhou, Jiangsu 215123 China; 6 Center for Integrative Genomics, University of Lausanne, Lausanne, Switzerland

**Keywords:** PGC-1α, Mitochondria, CD8, Memory, Anti-tumor immunity, Immunotherapy, CD8-positive T cells, Tumour immunology

## Abstract

Memory CD8 T cells can provide long-term protection against tumors, which depends on their enhanced proliferative capacity, self-renewal and unique metabolic rewiring to sustain cellular fitness. Specifically, memory CD8 T cells engage oxidative phosphorylation and fatty acid oxidation to fulfill their metabolic demands. In contrast, tumor-infiltrating lymphocytes (TILs) display severe metabolic defects, which may underlie their functional decline. Here, we show that overexpression of proliferator-activated receptor gamma coactivator 1-alpha (PGC-1α), the master regulator of mitochondrial biogenesis (MB), favors CD8 T cell central memory formation rather than resident memory generation. PGC-1α-overexpressing CD8 T cells persist and mediate more robust recall responses to bacterial infection or peptide vaccination. Importantly, CD8 T cells with enhanced PGC-1α expression provide stronger antitumor immunity in a mouse melanoma model. Moreover, TILs overexpressing PGC-1α maintain higher mitochondrial activity and improved expansion when rechallenged in a tumor-free host. Altogether, our findings indicate that enforcing mitochondrial biogenesis promotes CD8 T cell memory formation, metabolic fitness, and antitumor immunity in vivo.

## Introduction

CD8 T cells mount protective responses against viral/bacterial infections and cancers.^1,2^ The infiltration of cytotoxic CD8 T cells into tumors and their long-term persistence in vivo are positively correlated with an improvement in the disease-free survival of cancer patients.^3–5^ Memory CD8 T cells are characterized by the expression of CD62L and CD127, which allow their trafficking into lymphoid tissues and enhanced survival capacity, respectively.^6,7^ Metabolically, memory CD8 T cells display a considerable spare respiratory capacity (SRC), which is largely driven by fatty acid oxidation (FAO) compared to naive T cells having lower levels of oxidative phosphorylation (OXPHOS) or effector CD8 T cells, which favor glycolysis to support their effector function.^8^ In addition, a unique feature of memory T cells is their ability to mediate more rapid recall responses at a higher magnitude upon antigen rechallenge.^9,10^ Of note, CD8 memory T cells have more mitochondrial mass than their naive counterparts, supporting a metabolic phenotype that empowers memory T cells with superior metabolic fitness, including both increased OXPHOS and glycolytic capacity.^11^

Recently, great interest has emerged to potentiate the efficacy of cancer immunotherapy by modulating T cell metabolism.^12–22^ Peroxisome proliferator-activated receptor gamma coactivator 1-alpha (PGC-1α) is a key regulator of mitochondrial biogenesis (MB) and modulates OXPHOS and FAO, which are the cardinal metabolic features of T cell memory induction.^23^ Here, we demonstrate that CD8 T cells overexpressing PGC-1α show increased in vivo persistence and an enhanced recall response to *Listeria-Ova* infection or OVA peptide vaccination. Specifically, enforced PGC-1α expression promotes central memory T cell (Tcm) formation rather than tissue-resident memory (Trm) generation. In addition, PGC-1α-transduced CD8 T cells show sustained mitochondrial structural and functional fitness both in vitro and in vivo. Moreover, adoptive cell transfer (ACT) of CD8 T cells overexpressing PGC-1α leads to an improved antitumor response in melanoma-bearing mice. Importantly, tumor-infiltrating lymphocytes (TILs) overexpressing PGC-1α maintain higher mitochondrial activity and increased expansion capacity when rechallenged in tumor-free hosts. These observations might provide important clues for novel therapeutic interventions to enhance antitumor immunity by improving the metabolic fitness of T cells for ACT.

## Results

### **E**nforced PGC-1α expression promotes CD8 T cell persistence, memory formation, and antigen recall potential

Recent studies from our group and others showed that the deficiency of Rictor, the core component of mammalian target of rapamycin complex 2 (mTORC2), favors CD8 T cell memory differentiation through transcriptional and metabolic reprogramming, at least partly due to enhanced Foxo1 transcriptional activity.^24,25^ More specifically, we demonstrated that mTORC2 deficiency leads to increased PGC-1α expression and mitochondrial activity in CD8 T cells.^25^ Although PGC-1α is well characterized in various organs, such as the heart, skeletal muscle, or liver, its expression pattern and functionality in CD8 T cells remain elusive. To this end, we first measured the expression levels of PGC-1α and PGC-1β in naive CD8 T cells or at different time points post activation (24, 48, and 72 h) and upon restimulation (24, 48, and 72; Supplementary Fig. 1a). The mRNA level of PGC-1α in CD8 T cells is decreased at 24, 48, and 72 h post activation compared to naive T cells, and PGC-1β expression is comparable between naive and 24 h post activation, which is then downregulated at the other time points tested (Supplementary Fig. 1b, c). Furthermore, the mRNA levels of both PGC-1α and PGC-1β in CD8 T cells were downregulated upon T-cell receptor (TCR) restimulation (Supplementary Fig. 1d, e). However, PGC-1α and PGC-1β protein expression was elevated 24 or 48 h post activation (Supplementary Fig. 1f**)** and 72 h post restimulation in the case of PGC-1α, whereas PGC-1β protein expression remained unchanged (Supplementary Fig. 1g), indicating that certain posttranslational mechanisms may control the stability of these two proteins in CD8 T cells. In addition, we measured the expression levels of PGC-1α and β mRNA in CD8 T cells from the tumor bed and the spleen 14 days post tumor engraftment (Supplementary Fig. 1h). Interestingly, T cells isolated from the tumor site displayed much lower levels of PGC-1α and β expression than CD8 T cells recovered from the spleen (Supplementary Fig. 1i, j). This finding is in line with a previous study showing that PGC-1α is gradually decreased in TILs.^20^ Additionally, to further investigate the impact of chronic TCR stimulation on PGC-1α and β, we analyzed CD8 T cells 7 and 21 days post chronic Lymphocytic Choriomeningitis Virus (LCMV, clone 13) infection (Supplementary Fig. 1k). Of note, the expression levels of both PGC-1α and β were markedly enhanced 21 days post infection compared to day 7 (Supplementary Fig. 1l, m), highlighting a differential PGC-1α and β expression pattern in exhausted T cells during chronic infection or in the tumor microenvironment (TME).

To explore the precise role of PGC-1α in CD8 T cell memory formation and persistence, we transduced OT-1 T cells (CD8 T cells bearing TCRs specific for ovalbumin) with a retroviral-based system overexpressing PGC-1α or mock control vector (SCR), detected by a fluorescent molecule or Thy1.1 (Supplementary Fig. 2a). Following transduction, the relative PGC-1α mRNA levels were 80-fold higher in PGC-1α-overexpressing T cells than in untransduced (green fluorescent protein (GFP) negative) T cells (Supplementary Fig. 2b). In addition, PGC-1α overexpression led to the increased expression of several subunits of the electron transport chain (ETC), such as NDUFA8, NDUFS8 (complex I), Cox5a, Cox6C (complex IV), and ATP5G3 (complex V), at the mRNA level compared to SCR controls (Supplementary Fig. 2c). The expression levels of SOD2 and CPT2, which are involved in reactive oxygen species (ROS) detoxification and FAO, respectively, were also increased in PGC-1α-transduced T cells (Supplementary Fig. 2c). Moreover, PGC-1α overexpression in CD8 T cells led to a higher ratio of mitochondrial respiration to mitochondrial mass (Supplementary Fig. 2d) and increased mitochondrial mass (Supplementary Fig. 2e), mitochondrial respiration (Supplementary Fig. 2f), and mitochondrial membrane potential (Supplementary Fig. 2g) than SCR. Altogether, these observations suggest markedly enhanced mitochondrial activity with PGC-1α overexpression in CD8 T cells. Consistently, we observed the elevated mitochondrial ROS production in PGC-1α-engineered T cells (Supplementary Fig. 2h). However, total cellular ROS production remained comparable between SCR and PGC-1α-engineered T cells (Supplementary Fig. 2i). Furthermore, T cells with enforced PGC-1α expression upregulated their basal oxygen consumption rate (OCR) and responded with an increased SRC upon Carbonyl cyanide 4-(trifluoromethoxy)phenylhydrazone (FCCP) treatment, thereby showing an enhanced mitochondrial reserve as in memory T cells (Supplementary Fig. 2j–l). Thus, CD8 T cells overexpressing PGC-1α are largely protected from oxidative damage while harboring higher mitochondrial activity.

**Fig. 1:**
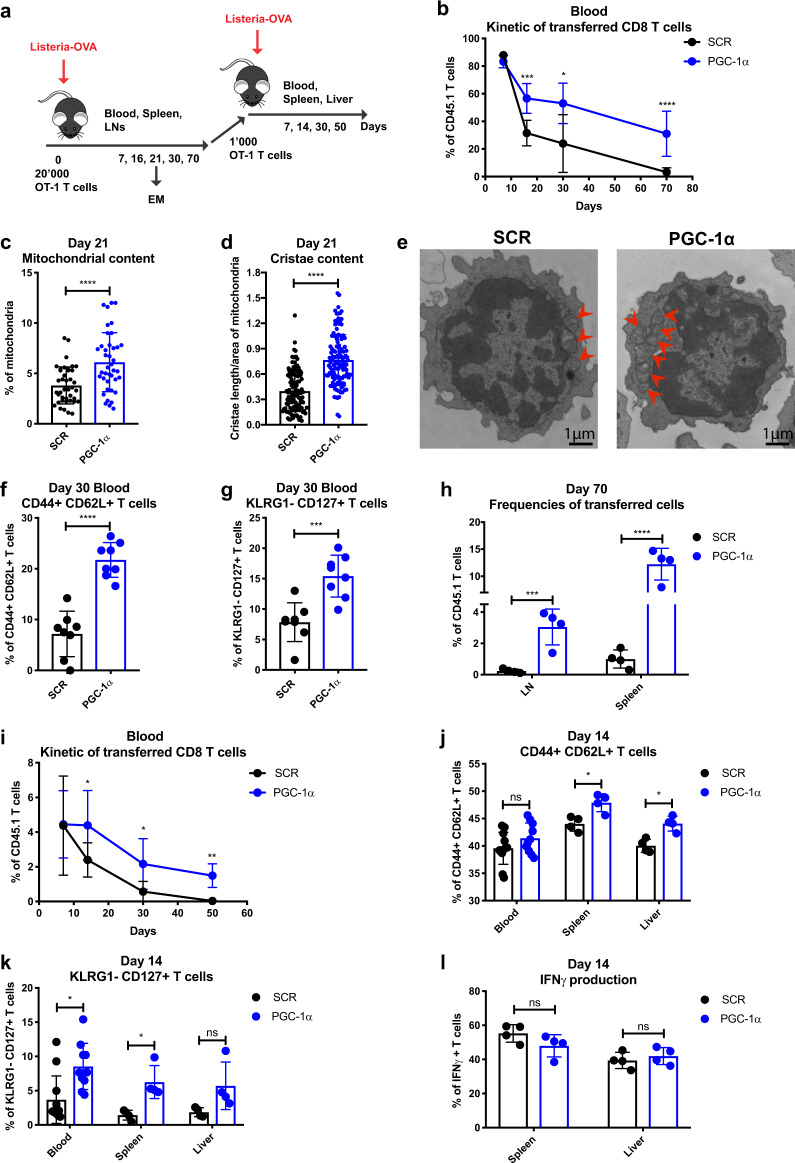
Enforced PGC-1α expression promotes CD8 T cell persistence, memory phenotype, and antigen recall potential. **a** Schematic representation of *Listeria-Ova* infection. A total of 20,000 CD45.1 OT-1 cells transduced with PGC-1α or SCR were transferred into CD45.2 naive recipients followed by 2000 CFU of *Listeria-Ova*. A total of 1000 transduced OT-1 cells were sorted 70 days post infection and transferred to CD45.2 naive hosts rechallenged with 2000 CFU of *Listeria-Ova*. **b** Kinetics of the adoptively transferred OT-1 cells in the blood post primary infection. **c** Percentage of mitochondria per cell area of transduced T cells at day 21 post infection. **d** Cristae length per area of mitochondria at day 21 post infection. **e** Representative examples of mitochondrial content per transduced cell. **f** Percentage in the blood of the CD44+ CD62L+ and **g** KLRG1− CD127+ populations at day 30 post primary infection. **h** Frequencies of transferred cells in LNs and spleen on day 70 post primary infection. **i** Kinetics of the adoptively transferred transduced OT-1 cells in the blood post rechallenge. **j** Percentage of CD44+ CD62L+ and **k** KLRG1− CD127+ populations in the blood, spleen, and liver on day 14 post rechallenge. **l** IFNγ production of transferred T cells in the spleen and liver on day 14 post rechallenge. **b**, **h**, **i** Gated on CD8+, **f**, **g**, **j**–**l** gated on CD8+ CD45.1+ GFP+. The results are representative of four independent experiments of primary infection and three independent experiments of rechallenge and represent the mean ± SD (4–10 mice per group). **c** *n* = 40 cells per group, and **d** *n* = 120 mitochondria per group. *p < 0.05; **p < 0.01; ***p < 0.001; ****p < 0.0001

To evaluate the functional profile of PGC-1α-overexpressing CD8 T cells in vivo, PGC-1α- or SCR-transduced CD45.1 OT-1 T cells were adoptively transferred into CD45.2 mice, followed by *Listeria-Ova* infection (Fig. 1a). PGC-1α-overexpressing OT-1 T cells persisted at higher levels in blood over time compared to SCR (Fig. 1b). To further confirm the effects of PGC-1α overexpression on mitochondrial structure or activity in vivo, the transferred cells were recovered from the spleens and examined by electron microscopy (EM) on day 21 post infection. Consistent with their in vitro phenotype, OT-1 T cells with enforced PGC-1α expression maintained increased mitochondrial (Fig. 1c) and cristae (Fig. 1d) content in vivo compared to their control counterparts (Fig. 1e). Importantly, at the contraction phase (day 16, data not shown) and at the early memory phase (day 30), an enhanced proportion of PGC-1α-overexpressing CD8 T cells in the blood exhibited a central memory phenotype, as characterized by the expression of CD44+ CD62L+ (Fig. 1f) and KLRG1− CD127+ (Fig. 1g). The increased frequency of PGC-1α-overexpressing T cells with the Tcm phenotype was further confirmed in spleens 30 days post infection (Supplementary Fig. 3a–g). In addition, significantly higher frequencies of PGC-1α-transduced T cells were present in the lymph nodes (LNs) and spleen at day 70 (Fig. 1h).

To better characterize the quality of memory T cells with enforced PGC-1α expression, we sorted CD45.1 mock or PGC-1α-transduced CD8 T cells 2 months after primary infection and adoptively transferred equal amounts of CD8 T cells into CD45.2 naive hosts followed by a secondary challenge. We clearly observed an improved persistence of PGC-1α-transduced T cells from day 14 to day 50 post rechallenge, illustrated by their higher frequency in the blood (Fig. 1i). Moreover, the enforced expression of PGC-1α promoted the central memory phenotype, as shown by the enhanced levels of CD44+ CD62L+ cells in the spleen and liver (Fig. 1j), and KLRG1− CD127+ cells in the spleen and blood (Fig. 1k). Nevertheless, the effector functions of the transferred cells were comparable in peptide restimulated PGC-1α and SCR-transduced OT-1 T cells, as seen by similar interferon γ (IFNγ; Fig. 1l), tumour necrosis factor α (TNFα), granzyme B, and interleukin-2 (IL-2) production (data not shown).

The improved persistence of PGC-1α-transduced OT-1 T cells was further confirmed in the OVA/CpG peptide vaccination model (Supplementary Fig. 4a), as shown by the increased frequency of transferred PGC-1α-overexpressing T cells at day 13 and day 30 post vaccination (Supplementary Fig. 4b). Next, these vaccinated mice were rechallenged with *Listeria-Ova* on day 38, which resulted in an enhanced recall response by T cells overexpressing PGC-1α, as indicated by increased fold re-expansion (Supplementary Fig. 4c). It is largely expected that the enforced PGC-1α expression may promote T cell persistence in vivo; however, we show for the first time that PGC-1α overexpression endows memory T cells with improved recall capacity, indicating that the quality of memory T cells can be augmented solely by increasing PGC-1α levels.

### Enforced PGC-1α expression does not favor resident memory T cell generation

**Fig. 2:**
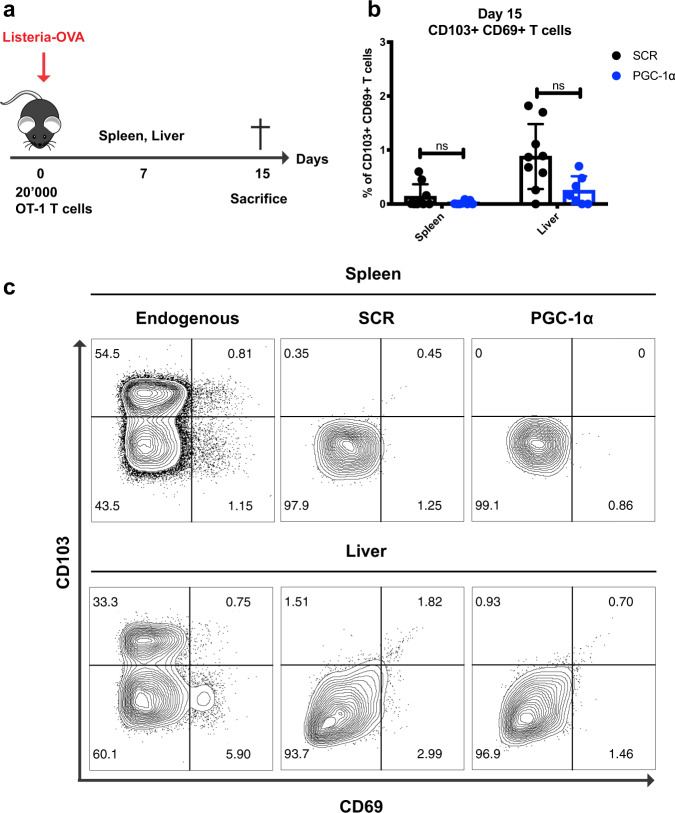
Enforced PGC-1α expression does not favor resident memory T cell generation. **a** Schematic representation of *Listeria-Ova* infection. A total of 20,000 CD45.1 OT-1 cells transduced with PGC-1α or SCR were transferred into CD45.2 naive recipients followed by 2000 CFU of *Listeria-Ova*. **b** Percentage of the CD103+ CD69+ population in the spleen and liver on day 15 post infection (pooled data from two experiments). **c** Representative histograms illustrating CD103 and CD69 expression in the endogenous T cell population and the transferred transduced OT-1 T cells from spleen and liver. **b**, **c** Gated on CD8+ CD45.1+ GFP+. Data are representative of two independent experiments and are presented as the mean ± SD (7–9 mice per group). *p < 0.05; **p < 0.01; ***p < 0.001; ****p < 0.0001

Trm cells are a subset of memory T cells characterized by the expression of CD103 and CD69 markers and by the absence of LN homing receptors. Trm cells localize in peripheral tissues, providing an early T cell response upon antigen recognition.^3^ Since PGC-1α overexpression enhanced Tcm formation, we tested whether enforced PGC-1α expression could promote the Trm generation, another long-lasting memory population. Nevertheless, our results indicated that T cells with higher expression of PGC-1α did not favor Trm cell formation post *Listeria-Ova* (Fig. 2a) or *Influenza-Ova* infection (data not shown). Indeed, SCR and PGC-1α-transduced OT-1 T cells showed comparable expression levels of the CD103 and CD69 markers at day 15 post *Listeria-Ova* infection in the spleen and liver (Fig. 2b, c).

### CD8 T cells overexpressing PGC-1α show enhanced metabolic fitness, improved persistence, and accumulation at the tumor site

**Fig. 3:**
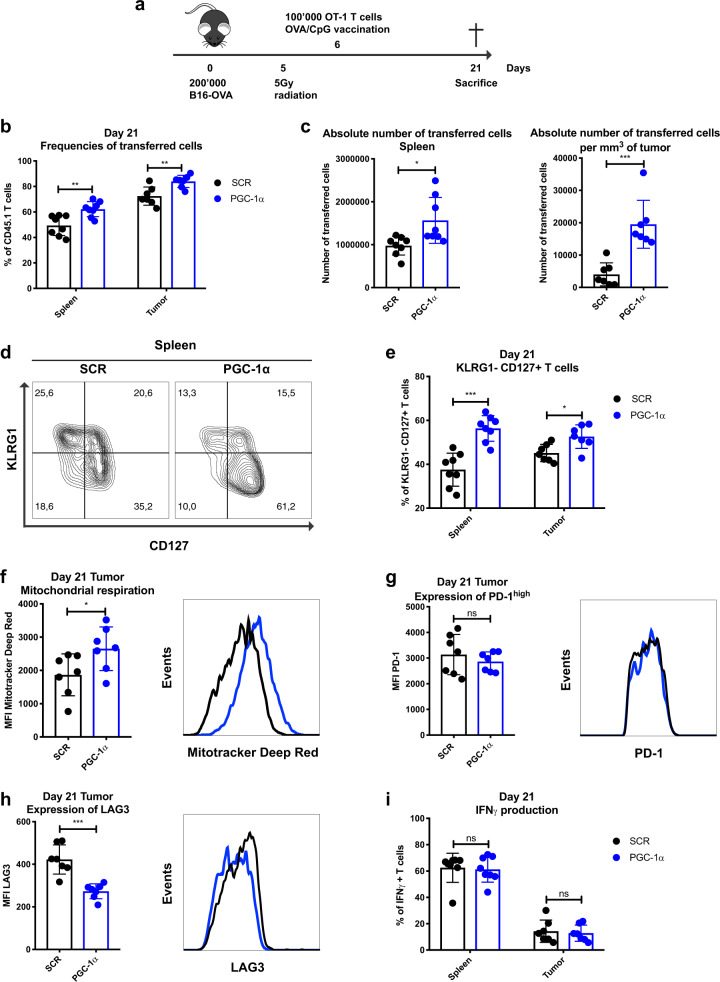
CD8 T cells overexpressing PGC-1α show enhanced metabolic fitness, improved persistence, and accumulation at the tumor site. **a** Schematic representation of the B16-OVA tumor model. CD45.2 mice were engrafted with 200,000 B16-OVA melanoma cells (s.c.), received 5 Gy whole body radiation and 100,000 CD45.1-transduced OT-1 T cells (i.v.) followed by OVA/CpG vaccination (s.c.). Mice were sacrificed on day 21. **b** Frequencies of transferred cells in the spleen and tumor (% of CD8+). **c** Absolute number of transferred cells in the spleen (left panel) and per mm^3^ of tumor (right panel). **d** Representative histograms illustrating KLRG1− CD127+ T cells in the spleen. **e** Percentage of the KLRG1− CD127+ population in spleen and tumor. **f** Mean Fluorescence Intensity (MFI) of MitoTracker Deep Red of TILs. **g** MFI of PD-1^high^ in TILs. **h** MFI of LAG3 in TILs. **i** IFNγ production of transferred T cells in spleen and tumor. **d**–**i** Gated on CD8+ CD45.1+ GFP+. Data are representative of three independent experiments and are presented as the mean ± SD (8 mice per group). *p < 0.05; **p < 0.01; ***p < 0.001; ****p < 0.0001

Next, we tested whether PGC-1α overexpression could promote CD8 T cell infiltration into the tumor due to their better persistence and metabolic fitness, leading to an improved antitumor response. We thus adoptively transferred SCR or PGC-1α-transduced OT-1 T cells into B16-OVA tumor-bearing mice on day 6 post tumor graft and evaluated the phenotype of the transferred cells at day 21 (Fig. 3a). The frequency of PGC-1α-overexpressing T cells was significantly enhanced both in terms of relative percentages in the periphery and at the tumor site (Fig. 3b), and of absolute number of PGC-1α-transduced OT-1 T cells in the spleen and per mm^3^ of tumor (Fig. 3c). Similar to infection settings, OT-1 T cells overexpressing PGC-1α showed the phenotypic hallmarks of memory T cells, as illustrated by a higher percentage of KLRG1− CD127+ cells in the periphery and at the tumor site compared to SCR (Fig. 3d, e). Furthermore, the increased mitochondrial respiration capacity in OT-1 T cells overexpressing PGC-1α was also maintained in the TME (Fig. 3f), suggesting that enhanced metabolic fitness might be achieved via PGC-1α-mediated improved mitochondrial activity. It is well established that tumor antigen-specific T cells gradually acquire an exhausted phenotype within the TME, which is characterized by upregulation of coinhibitory molecules, including PD-1 and LAG3, and a concomitant decline in cellular proliferative capacity and functionality.^26^ Although PD-1 expression remained similar in both T cell populations (Fig. 3g), the expression of the inhibitory receptor LAG3 (Fig. 3h) was significantly lower in T cells with enforced PGC-1α expression compared to their SCR counterpart. This decreased LAG3 expression might suggest a less exhausted phenotype of PGC-1α-overexpressing CD8 T cells. Nevertheless, the effector cytokine production capacity post peptide restimulation in the periphery and at the tumor site was comparable between the SCR and PGC-1α overexpression groups in terms of IFNγ (Fig. 3i), TNFα, granzyme B, and IL-2 production (data not shown). Collectively, our findings demonstrate that higher PGC-1α expression in OT-1 cells increases mitochondrial respiration and confers a metabolic advantage, resulting in enhanced in vivo persistence and accumulation at the tumor site.

### Enforced PGC-1α expression in T cells boosts antitumor immunity

**Fig. 4:**
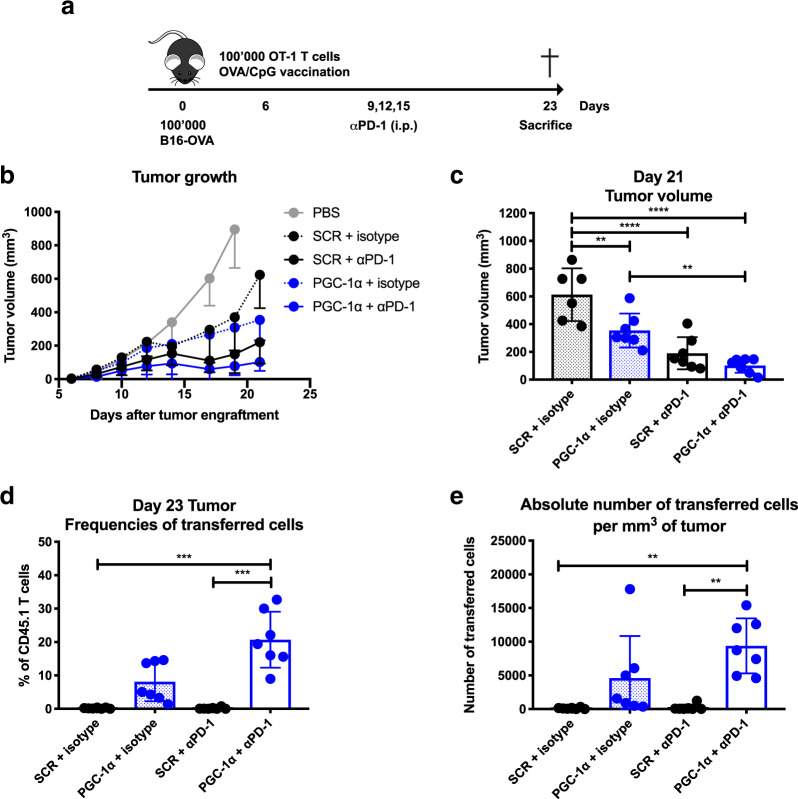
Enforced PGC-1α expression in T cells boosts antitumor immunity. **a** Schematic representation of the experimental setup for the tumor growth assay. CD45.2 mice were engrafted with 100,000 B16-OVA (s.c.), received 100,000 CD45.1-transduced OT-1 cells (i.v.), followed by OVA/CpG vaccination (s.c.). Either αPD-1 or isotype control was administered (i.p.) on days 9, 12, and 15 post engraftment. Tumor growth was measured from T cell transfer until day 21. **b** Tumor growth. **c** Tumor volume at day 21. **d** Frequencies of transferred cells at the tumor site (% of CD8+). **e** Absolute number of transferred cells per mm^3^ of tumor. Data are representative of three independent experiments and are presented as the mean ± SD (6–7 mice per group). *p < 0.05; **p < 0.01; ***p < 0.001; ****p < 0.0001

Exhausted T cells can be reinvigorated by αPD-1 blockade, leading to improved T cell functionality and antitumor response in a considerable proportion of cancer patients.^27^ Given the metabolic advantages conferred by PGC-1α expression, we then tested the therapeutic benefit of combining ACT of PGC-1α-engineered CD8 T cells with PD-1 blockade. To this end, 6 days post B16-OVA tumor engraftment, mice received PGC-1α or SCR-transduced OT-1 T cells followed by OVA/CpG vaccination and αPD-1 treatment (Fig. 4a). Importantly, PGC-1α-overexpressing CD8 T cells significantly delayed the tumor growth compared to SCR when treated with isotype control (Fig. 4b, c). Nevertheless, OT-1 T cells overexpressing PGC-1α only tended to further improve tumor control when combined with αPD-1 compared to SCR (Fig. 4b, c). In addition, we did observe certain tumor controls with αPD-1 treatment alone in our model with both T cell populations compared to their isotype control (Fig. 4b, c). Consistently, we observed higher proportions (Fig. 4d) and absolute numbers (Fig. 4e) of OT-1 T cells overexpressing PGC-1α present at the tumor site, which tended to be further potentiated when combined with αPD-1 treatment. Our results demonstrate that enforced PGC-1α expression in CD8 T cells could boost antitumor immunity, but fails to display a significant synergy with αPD-1 treatment.

### TILs overexpressing PGC-1α show better recall capacity and differentiate into memory T cells with enhanced metabolic fitness

**Fig. 5:**
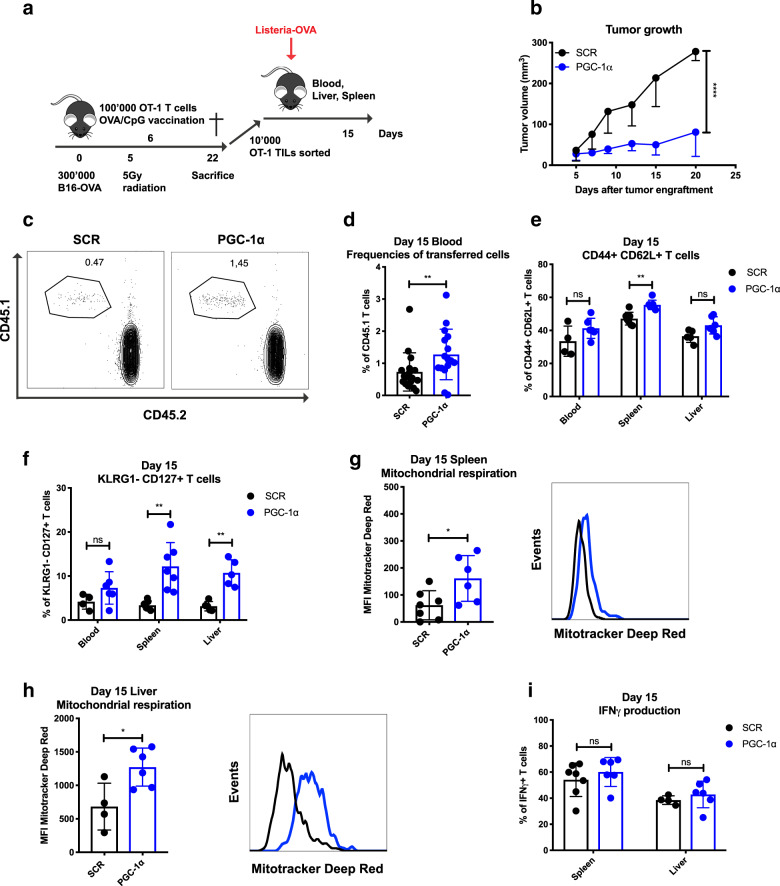
TILs overexpressing PGC-1α show better recall capacity and differentiate into memory T cells with enhanced metabolic fitness. **a** Schematic representation of the experimental setup for TIL retransfer in naive hosts. CD45.2 mice were engrafted with 300,000 B16-OVA (s.c.), received 5 Gy whole body irradiation and 100,000 CD45.1-transduced OT-1 cells (i.v.), followed by OVA/CpG vaccination (s.c.). TILs were sorted 22 days post tumor engraftment, and 10,000 cells were retransferred in CD45.2 naive hosts (i.v.) and challenged with *Listeria-Ova* 2000 CFU (i.v.). Analysis was performed 15 days post recall. **b** Tumor growth. **c** Representative dot plots illustrating the frequencies of transferred cells. **d** Frequencies of transferred cells (pooled data from two experiments). **e** Percentage of CD44+ CD62L+ and **f** KLRG1− CD127+ populations in the blood, spleen, and liver. **g** MFI of MitoTracker Deep Red of transferred cells in spleen and **h** in liver. **i** IFNγ production of transferred T cells in the spleen and liver. **c**, **d** Gated on CD8+, and **e**–**i** gated on CD8+ CD45.1+ GFP+. Data are representative of two independent experiments and are presented as the mean ± SD (7–9 mice per group). If <100 transferred cells were detected by flow cytometry, the mouse was removed from the analysis. *p < 0.05; **p < 0.01; ***p < 0.001; ****p < 0.0001

To further characterize whether the enhanced metabolic fitness upon PGC-1α overexpression could be maintained over time, PGC-1α- or SCR-transduced OT-1 T cells were transferred into tumor-bearing mice and expanded upon OVA/CpG vaccination (Fig. 5a). Confirming our previous experiments, ACT of T cells overexpressing PGC-1α led to better tumor control than its SCR counterpart (Fig. 5b). Additionally, PGC-1α- or SCR-transduced TILs were then recovered from the tumors (day 22), isolated as highly purified cells by flow cytometry-based sorting and retransferred into naive hosts, followed by *Listeria-Ova* infection. The re-expansion capacity of tumor-exposed PGC-1α or SCR-transduced T cells was monitored in the blood, liver, and spleen (day 15; Fig. 5a). TILs overexpressing PGC-1α generated more secondary effector CD8 T cells in the blood at day 15 (Fig. 5c, d). Moreover, TILs with enforced expression of PGC-1α displayed cardinal phenotypic features of memory T cells, as revealed by an increase in the percentage of CD44+ CD62L+ cells observed in the spleen (Fig. 5e), and of KLRG1− CD127+ cells in both spleen and liver (Fig. 5f). Metabolically, CD8 T cells overexpressing PGC-1α maintained their enhanced mitochondrial activity upon rechallenge in the spleen and the liver (Fig. 5g, h). Nevertheless, their functionality, illustrated by IFNγ production, was not further improved (Fig. 5i). Thus, tumor-exposed PGC-1α-overexpressing CD8 T cells are able to maintain their mitochondrial content/activity, and this programmed metabolic advantage bestows them with more robust re-expansion capacity in response to antigen recall in a tumor-free microenvironment.

## Discussion

Memory CD8 T cells can persist in the absence of antigen and harbor a unique metabolic machinery to sustain their cellular fitness, which provides long-term protection against tumors. In the present study, we demonstrated that overexpression of PGC-1α, the key regulator of MB, in mouse CD8 T cells led to sustained metabolic fitness for their long-lasting survival and persistence in vivo during acute infection or at the tumor site.

Interestingly, PGC-1α expression is dynamically and tightly regulated during T cell activation or functional differentiation. We confirmed that TILs show much decreased PGC-1α expression at the tumor site as previously described,^20^ whereas there was an upregulation of PGC-1α 21 days post chronic infection. Along the same lines, it was shown that T cells displayed enhanced mitochondrial mass but decreased respiratory ability and a higher percentage of depolarized mitochondria during chronic infection (clone 13, LCMV) compared to acute infection (clone Armstrong, LCMV),^21^ indicating the accumulation of dysfunctional mitochondria in exhausted T cells driven by chronic infection. Thus, the key regulatory factors driving the differential expression pattern of PGC-1α and mitochondrial defects in CD8 T cells during chronic viral infection and at the tumor site remain to be assessed in detail. Whether T cells overexpressing PGC-1α are more resistant to a highly immunosuppressive and metabolically stressed TME or chronic infection deserves further scrutiny. Moreover, we found that PGC-1α stayed mainly in the nucleus, whereas PGC-1β was exclusively expressed in the cytoplasm of CD8 T cells (data not shown), suggesting that they might mediate distinct biological functions.

Notably, enforced PGC-1α expression in CD8 T cells specifically promoted Tcm generation rather than Trm formation post *Listeria-Ova* or *Influenza-Ova* infection. Consistently, this result is in line with a recent study showing that Trm cells rely more on extracellular fatty acid uptake for their long-term survival compared to Tcm cells, thus highlighting different metabolic needs for distinct memory subsets,^28^ which further supports the notion that PGC-1α expression specifically favored Tcm formation.

One unique feature of memory T cells is to “remember” the pathogen they have encountered and mediate a robust recall response very rapidly after antigen rechallenge. In particular, CD8 memory T cells harbor greater mitochondrial mass than naive T cells.^11^ In comparison to primary effector T cells, secondary effector T cells show enhanced proliferative capacity, produce more cytokines and maintain higher ATP levels upon activation.^9,10^ Interestingly, the recall response of memory CD8 T cells could be augmented solely by increasing PGC-1α expression. Interestingly, despite harboring an enhanced mitochondrial mass and activity, PGC-1α-transduced T cells showed relatively higher mitochondrial ROS but comparable total ROS production compared to the SCR control. This finding may suggest a parallel increase in antioxidant enzyme production to protect PGC-1α-overexpressing cells from oxidative stress,^29^ and we did observe a higher level of SOD2 expression associated with PGC-1α overexpression. Importantly, PGC-1α-transduced TILs were able to maintain a stable metabolic advantage in vivo and expanded to a greater extent in response to antigen recall in a tumor-free microenvironment. Therefore, small compounds known to improve MB and mitochondrial activity, such as resveratrol, might be used to favor CD8 T cell persistence and boost cancer immunotherapy. A detailed understanding of MB-modulating drugs may help to shed light on the mechanisms underlying memory T cell differentiation and provide new clues for therapeutic interventions in immunotherapy of cancer.

One recent study showed that TILs have chronic Akt activity and gradually lose mitochondrial mass, which was accompanied by reduced PGC-1α expression.^20^ Indeed, Foxo1 has been reported to regulate PGC-1α transcription in muscle cells by directly binding to the promoter and stimulating PGC-1α expression. Conversely, insulin abrogates PGC-1α transcription by activating Akt, which phosphorylates and thus inhibits Foxo1 transcriptional activity.^30^ Along the same lines, we observed higher mRNA levels of PGC-1α in CD8 T cells activated in the presence of an Akt inhibitor (data not shown); however, it remains unclear whether Foxo1 directly controls PGC-1α transcription in CD8 T cells. In the present study, we observed increased therapeutic power of T cells overexpressing PGC-1α. These effects were based on their enhanced persistence and metabolic fitness, which provided higher numbers of secondary effector T cells to target tumor cells. Nevertheless, in addition to the expression level, PGC-1α activity is also regulated via posttranslational modifications, including phosphorylation and acetylation/deacetylation in muscle and liver.^23^ Further investigation is required to determine whether those posttranslational modifications of PGC-1α could also impact CD8 T cell fitness and antitumor immunity.

Interestingly, αPD-1 treatment has been shown to enhance mitochondrial respiration in CD8 T cells in certain tumor models,^31^ which is consistent with the findings that PD-1 signaling blocks PGC-1α expression and mitochondrial activity.^21^ However, enforcing PGC-1α expression in CD8 T cells only tended to have an additive effect with checkpoint blockade to boost antitumor immunity. Clearly, further studies are required to characterize the sequence of the combination treatment with maximal therapeutic efficacy.

In conclusion, we demonstrated that PGC-1α overexpression improved the antitumor effects of ACT therapy by enhancing CD8 T cell persistence potential and metabolic fitness. However, no specific drugs activating PGC-1α are currently commercially available. Therefore, the development of novel strategies to improve the metabolic fitness of adoptively transferred T cells could promote the therapeutic efficacy of antitumor immunity. In addition, it remains to be investigated whether short-term stimulation of PGC-1α in TILs could boost their mitochondrial activity and long-lasting capacity, which may lead to improved tumor control upon reinfusion to the host.

## Materials and methods

### Animals

CD45.1 OT-1 mice were maintained at the University of Lausanne’s Specific Pathogen Free facility. CD45.2 C57BL/6 mice were purchased from Envigo. The present study was approved by the Veterinary Authority of the Canton Vaud (Permit #2688.1), and all experiments were performed in accordance with Swiss ethical guidelines.

### Transfection and retroviral transduction

We designed two retroviral constructs to overexpress PGC-1α in CD8 T cells. PGC-1α was under the control of the LTR promoter, and its expression was tracked by GFP or Thy1.1. The SCR control was our mock transduction vector control. Briefly, a scrambled (SCR) short hairpin RNA was expressed under the control of the H1 promoter, and a reporter gene, either the Zoanthus sp GFP (Zsgreen) molecule or Thy1.1, was cloned downstream of the hPGK promoter. PGC-1α-Thy1.1 or SCR-Thy1.1 were used only in Supplementary Fig. 2 to characterize mitochondrial mass stained with MitoTracker Green.

Transfection was performed according to the instructions of Turbofect Transfection Reagents (ThermoFisher Scientific) and as previously reported.^32^ Briefly, virus supernatant was collected and ultracentrifuged at 4 °C for 2 h. The spin infection was carried out at 2000×*g* at 32 °C for 90 min in a retronectin (Takara Clonotech)-coated plate. To stimulate the sorted OT-1 cells, we used CD3/CD28 Dynabeads (Gibco) at a 2:1 ratio. Twenty-four hours after stimulation, OT-1 cells were transferred to retronectin-coated plates with concentrated retrovirus. The transduced cells were maintained in vitro with recombinant human IL-2 (50 U/ml) from days 1 to 3, IL-2 (10 U/ml), recombinant human IL-7 (10 ng/ml), and recombinant human IL-15 (10 ng/ml) from days 4 to 5. Beginning on day 6 post activation, the medium was supplemented only with IL-7 (10 ng/ml) and IL-15 (10 ng/ml).

### CD8 T cell activation and restimulation

OT-1 T cells were isolated from the spleens of OT-1 mice. CD8 T cells were purified by the Mouse CD8 Naive T cell Isolated Kit (MojoSort^TM^, cat no. 480044). CD8 T cells (1 × 106 cells/mL) were plated into 24-well plates with 2 mL per well. Naive CD8 T cells were activated by treatment with αCD3 antibody (2 μg/mL, Invitrogen, 16-0031-86), αCD28 antibody (1 μg/mL, Invitrogen, 16-0281-86), and IL-2 (10 ng/mL, Pepro Tech, AF-200-02-1000) concurrently. Protein and mRNA levels of PGC-1α and PGC-1β were measured by western blot and reverse transcription polymerase chain reaction (RT-PCR), respectively, at 24, 48, and 72 h time points post activation. CD8 T cells were then continuously cultured with IL-2 (10 ng/mL) during a resting phase of 10 days. For restimulation, live activated CD8 T cells were collected by Ficoll-Paque (GE Healthcare, 17-1440-03), plated into 24-well plates at a concentration of 1 × 10^6^ cells/mL and reactivated as during initial activation. Finally, CD8 T cells were purified by Ficoll-Paque, and the protein and mRNA levels of PGC-1α and PGC-1β were measured by western blot and RT-PCR, respectively, at 24, 48, and 72 h time points post restimulation.

### Cell sorting

Sorting for PGC-1α or SCR-transduced cells was carried out by Live-Aqua Dead-, antimouse CD8-APC + (Ly2), antimouse CD45.1-Percyp-Cy5.5+ (A20), and GFP+ expression.

### OT-1 cell transfer and *Listeria-Ova* infection

Sorted PGC-1α or SCR-transduced OT-1 cells were transferred into naive host intravenously (i.v). Within 5 h after OT-1 cell transfer, 2000 colony-forming unit (CFU) of *Listeria-Ova* were administered i.v.

### P14 cell transfer, LCMV chronic infection (clone 13), and cell sorting

Five thousand activated or naive P14 cells were injected into naive hosts (i.v.), which were infected (i.v.) with 2 × 10^6^ pfu of LCMV (clone 13). The effector CD8 T cells (CD44+ CD62L−) were collected from the spleen at days 7 and 21 post infection.

### Memory T cell transfer and antigen rechallenge

At the indicated time points after primary infection, memory CD8 T cells were first enriched with the Mouse CD8 T Cell Isolation Kit (Stemcell Technology) and then processed for FACS sorting with congenic markers and GFP expression. A total of 1000 sorted CD45.1+ GFP+ CD8 T cells were transferred into the naive host, followed by *Listeria-Ova* rechallenge.

### Melanoma tumor models

B16-OVA tumor cells (105 or 3 × 105) were subcutaneously (s.c.) engrafted onto the flanks of C57BL/6 mice. If applied, 5 Gy whole body radiation was performed 5 days post tumor engraftment. One day later, 10^5^ OT-1 were adoptively transferred i.v. and mice were vaccinated (s.c.) with 10 µg SIINFEKL (N4) peptide (Protein and Peptide Chemistry Facility, UNIL) and 50 µg CpG (ODN 1826, U133-L01A, Trilink Biotechnologies). Tumors were measured with a caliber every 2–3 days from day 5 or 6. Spleens and tumors were harvested at the indicated time points post tumor engraftment. Tumors were dissociated with the Tumor Dissociation Kit (Milenyi Biotec) following the manufacturer’s instructions.

### Immune checkpoint blockade

B16-OVA tumor-bearing mice received 200 μg of αPD-1 (clone RMP-1–14, Rat IgG2a, BioXCell) or 2A3 isotype control (Rat IgG2a, BioXCell) on days 9, 12, and 15 post tumor engraftments.

### Flow cytometry

Transduced T cells overexpressing PGC-1α-Thy1.1 or expressing the mock control were stained 7 days post in vitro activation by MitoTracker Green (M7514 ThermoFisher), MitoTracker Deep Red (M22426, ThermoFisher), and TMRM (T668, ThermoFisher) for 30 min at 37 °C, and MitoSOX Red (M36008, ThermoFisher) and H2-DCFDA (D399, ThermoFisher) for 10 min at 37 °C.

Lymphocytes from blood, LNs, spleen, liver, and tumor were isolated and stained with antibodies obtained from BD Pharmingen (San Diego, CA, USA) and eBioscience (San Diego, CA, USA). For viability staining, cells were washed with phosphate-buffered saline and stained with LIVE/DEAD Fixable dye Aqua Dead (L34957, ThermoFisher) for 20 min at 4 °C. For cell surface staining, the following antimouse antibodies were used for 25 min at 4 °C: antimouse CD45.1-BV650 (A20), antimouse CD45.2-Pacific Blue (104), antimouse CD8α**-**PE-Texas Red (Ly2), antimouse CD62L-Percyp-Cy5.5 (MEL-14), antimouse CD44-APC-eFluo780 (IM7), antimouse KLRG1-PE-Cy7 (MAFA), antimouse CD127-PE (SB/199), antimouse PD-1-APC (J43), antimouse LAG3-PE (C9B7W), antimouse CD103-PE (2E7), and antimouse CD69-PE-Cy7 (H1.2F3). For intracellular cytokine staining, cells were fixed for 20 min at 4 °C and permeabilized with permeabilization wash buffer (421002, Biolegend) according to the manufacturer’s instructions. Cells were then stained with antimouse IFNγ-PerCp-Cy5.5 (XM61.2), antimouse TNFα-Pacific Blue (MP6-XT22), antimouse IL-2-PE (JES6-SH4), and antihuman/mouse Granzyme B-PE-Texas Red (GB11) for 30 min at 4 °C. Samples were resuspended in FACS buffer and acquired using an LSR-II flow cytometer (Becton-Dickinson, San Jose, CA) and analyzed using FlowJo software (Tree Star, Ashland, OR).

### In vitro restimulation

At indicated time points (infection or tumor-bearing conditions), lymphocytes from the spleen, liver or tumor were harvested, and plated into 96-well plates. The cells were thus incubated with 10 μM SIINFEKL peptide or PMA plus ionomycin for 30 min, followed by another 4 h of restimulation in the presence of Golgistop (BD) and Golgiplug (BD).

### SDS–PAGE and western blotting

Naive or CD8 T cells at defined time points post activation or restimulation were lysed in lysis buffer (20 mM Tris, pH 7.4, 2 mM EGTA, 1% NP-40, protease inhibitors). Equivalent amounts of proteins (20 g) were subjected to sodium dodecyl sulfate–polyacrylamide gel electrophoresis (SDS–PAGE) and transferred to nitrocellulose membranes. Membranes were then probed with the indicated primary antibodies targeting β-actin (AC026, ABclonal), PGC-1α (AB3242, Merck Millipore), or PGC-1β (ab176328, ABCAM), followed by the appropriate horseradish peroxidase-conjugated secondary antibodies (KPL).

### Real-time PCR

mRNA from in vitro cultured cells or ex vivo was purified using the RNeasy Plus Mini Kit (Qiagen). Complementary cDNA was generated by using SuperScript III reverse transcriptase (Life Technologies) and random primers (Invitrogen). Gene expression was assessed by quantitative PCR (qPCR) using SYBR green amplification detection reagent (Applied Biosystems) on a 7500 FAST Real-Time PCR System (Applied Biosystems). Ct values for each analyzed gene were normalized to β-2 microglobulin and adjusted for amplification efficiency. Primers to detect β2 M: (FW) 5′-AGACTGATACATACGCCTGCAG-3′, (RV) 5’GCAGGTTCAAATGAATCTTCAG-3′, PGC-1α: (FW) 5′-CCCTGCCATTGTTAAGACC-3′, (RV) 5′-TGCTGCTGTTCCTGTTTTC-3′,

PGC-1β: (FW) 5′-ACT ATG ATC CCA CGT CTG AAG AGT C-3′, (RV)

CCT TGT CTG AGG TAT TGA GGT ATT C-3′,

NDUFA8: (FW) 5′-TCAACGGTCGTGCGCTGAACTT-3′, (RV) 5′-CACACTGGTCAAACTTTGCCTGC,

NDUFS8: (FW) 5′-TGGCGGCAACGTACAAGTAT-3′, (RV) 5′-CCTCGGATGAGTTCTGTCCA-3′,

Cox5a: (FW) 5′-TTGATGCCTGGGAATTGCGTAAAG-3′, (RV) 5′- AACAACCTCCAAGATGCGAACAG-3′,

Cox6c: (FW) 5′-GGAGTTGCCGCTGCCTATAA-3′, (RV) 5′-AATTCTGCATACGCCTTCTTTCTT-3′,

ATP5G3: (FW) 5′-GTAGGAGTTGCTGGTTCTGGTG-3′, (RV) 5′-GCTTCAGACAAGGCAAATCCCAG-3′,

SOD2: (FW) 5′-GGCCAAGGGAGATGTTACAA-3′, (RV) 5′-GAACCTTGGACTCCCACA-3′,

CPT2: (FW) 5′-GGCCAGGGCTTTGACCGACACT-3′, (RV) 5′-TGTCAAAGCCATCAGGACCAC-3′.

### Seahorse analysis

For analysis of the metabolic phenotype of CD8 T cells, OCR (in pmol/min) was measured using the Seahorse XF-96 metabolic extracellular flux analyzer (Seahorse Bioscience). Transduced T cells sorted day 7 post activation were rested overnight before performing a Seahorse assay. CD8 T cells were washed and resuspended in DMEM (Sigma) containing 10 mM glucose (Sigma) and 2 mM glutamine (Life Technology). The cells were then plated onto Seahorse cell plates (3 × 10^5^ cells per well) coated with Cell-Tak (BD Bioscience) to allow for their attachment. Perturbation profiling of substrate use by CD8 T cells was achieved by the addition of oligomycin (2 µM), FCCP (2 μM), rotenone (0.5 µM), and antimycin A (0.5 µM; all from Sigma-Aldrich).

### Electron microscopy

Electron microscope images were taken with a Philips CM100 transmission electron microscope at an acceleration of 80 kV with a TVIPS TemCam-F416 digital camera. Images were taken at a magnification of 4800× and 11,000×. The software used was EMMENU, and images were exported in 8 bits. Samples were prepared by a fixation step with glutaraldehyde 2.5% (EMS) and osmium tetroxide 1% (EMS). After several washes with water and acetone (Sigma), samples were embedded in Epon (Sigma) resin. Fifty-nanometer slides were prepared using a Leica Ultracut microtome. Before imaging, slides were also contrasted using uranyl acetate (Sigma) and Reynolds lead citrate (Sigma). The analysis of the images was performed blinded using 3dmod (University of Colorado) and Fiji (ImageJ) software. Briefly, to determine the percentage of mitochondria per transduced cell, a grid was applied, and each intersection was defined as part of the nucleus, cytoplasm, or mitochondria. To quantify the cristae content, the length of each cristae was measured divided by the mitochondrial area.

### Statistical analysis

All tests were performed using GraphPad Prism software (La Jolla, CA). All data are presented as the mean + SD. Normality of the data was tested with a Shapiro–Wilk test. Comparisons between two unpaired groups were performed by parametric Student’s *t*-test or nonparametric Mann–Whitney test. For multiple comparisons, a multiple *t*-test was applied (correction with the Holm–Sidak method). For multiple comparisons, a parametric one-way analysis of variance (ANOVA) or nonparametric Krustal–Wallis test was performed (correction with Dunn’s multiple comparisons test). For multiple comparisons involving different groups at diverse time points, a parametric two-way ANOVA was performed (corrected with Sidak’s multiple comparisons test). *p* < 0.05 were considered statistically significant (*p < 0.05; **p < 0.01; ***p < 0.001). All results are representative of at least two independent experiments.

## Supplementary information

Supplementary Infirmation

Supplementary Figure 1

Supplementary Figure 2

Supplementary Figure 3

Supplementary Figure 4
