# Supplementary material for: Enforced PGC-1α expression promotes CD8 T cell fitness, memory formation and antitumor immunity
Source: Cell Mol Immunol. 2020 Feb 13;18(7):1761–71. doi: 10.1038/s41423-020-0365-3 (PMC8245409; doi:10.1038/s41423-020-0365-3)
Supplement: Supplementary file 1 — Supplementary Infirmation [file 41423_2020_365_MOESM1_ESM.docx]

**Supplementary Figure 1**

**(A)** Schematic representation of the experimental setup for *in vitro* analysis upon T cell activation, resting phase and restimulation. **(B)** Expression of PGC-1α at the mRNA level upon activation. **(C)** Expression of PGC-1β at the mRNA level upon activation. (**D**) Expression of PGC-1α at the mRNA level upon restimulation. (**E**) Expression of PGC-1β at the mRNA level upon restimulation. (**F**) Expression of PGC-1α and PGC-1β at the protein level upon activation. (**G**) Expression of PGC-1α and PGC-1β at the protein level upon restimulation. **(H)** Schematic representation of the experimental setup for the tumor growth assay. CD45.2 mice were engrafted with 100,000 or 200,000 B16-OVA (s.c.), received 100,000 CD45.1 OT-1 cells (i.v.), followed by OVA/CpG vaccination (s.c.). The effector cells were sorted 14 days post-tumor engraftment from the spleen and tumor. **(I)** Expression of PGC-1α at the mRNA level in effector cells and TILs 14 days post-tumor engraftment (pooled data of two independent experiments). **(J)** Expression of PGC-1β at the mRNA level in effector cells and TILs 14 days post-tumor engraftment (pooled data of two independent experiments). **(K)** Schematic representation of the LCMV clone 13 infection model. A total of 5,000 P14 CD45.1 CD8 T cells were transferred to naïve CD45.2 mice followed by 2x10^6^ pfu of clone 13 LCMV infection one day later. The effector CD8 T cells were sorted 7 and 21 days post chronic infection. **(L)** Expression of PGC-1α at the mRNA level 7 and 21 days post chronic infection (pooled data of two independent experiments). **(M)** Expression of PGC-1β at the mRNA level 7 and 21 days post chronic infection (pooled data of two independent experiments). Data are representative of at least two experiments and are presented as the mean ± SD (**B-C** 5 mice per group), (**D-E** 3 mice per group), (**F-G** 3 mice per group), (**I-J** 5 mice for effector T cell analysis, 8 mice for TIL analysis), (**L-M** 10 mice at day 7 and 9 mice at day 21). If PGC-1α or PGC-1β levels remained undetermined on the 7500 FAST Real-Time PCR System, the mouse was removed from the analysis. *, p < 0.05; **, p < 0.01; ***, p < 0.001; ****, p < 0.0001.

**Supplementary Figure 2**

**(A)** Schematic representation of the experimental setup for *in vitro* analysis 7 days post transduction. **(B)** mRNA level of PGC-1α in sorted transduced OT-1 T cells. **(C)** mRNA levels of NDUFA8, NDUFS8 (complex I), Cox5a, Cox6c (complex IV) ATP5G3 (complex V), SOD2 and CPT2 in sorted transduced OT-1 T cells. (**D**) Ratio of MitoTracker Deep Red on MitoTracker Green. **(E)** MFI of MitoTracker Green. **(F)** MFI of MitoTracker Deep Red. **(G)** MFI of TMRM. **(H)** MFI of MitoSOX Red (pooled data from 4 experiments). **(I)** MFI of CM H_2_-DCFDA (pooled data from 3 experiments). (**J**) Oxygen consumption rate (OCR) measured by Seahorse analysis. **(K)** Basal OCR. **(L)** Spare respiratory capacity (SRC). **(D-I)** gated on CD8+ GFP+. Data are representative of at least two independent experiments and are presented as the mean ± SD, (**B**) 4 mice per group, (**C**) 6 mice per group, (**D-G**) 3 mice per group, (**H**) 12 mice per group, (**I**) 9 mice per group, (**J-L**) 7 mice per group. *, p < 0.05; **, p < 0.01; ***, p < 0.001; ****, p < 0.0001.

**Supplementary Figure 3**

**(A)** Schematic representation of the *Listeria-Ova* infection model. A total of 50,000 CD45.1 OT-1 cells transduced with PGC-1α or SCR were transferred into CD45.2 naïve recipients followed by 2,000 CFU of *Listeria-Ova* for analysis of the spleen at day 30 post infection. **(B)** Frequency of transferred cells in the spleen. **(C)** MFI of MitoTracker Deep Red. **(D)** Representative histograms of the CD44+ CD62L+ population. **(E)** Percentage of the CD44+ CD62L+ population. **(F)** Representative histograms of the KLRG1- CD127+ population. **(G)** Percentage of the KLRG1- CD127+ population. **(B)** Gated on CD8+, **(C-G)** gated on CD8+ CD45.1+ GFP+. Data are representative of two independent experiments and are presented as the mean ± SD (8 mice per group). *, p < 0.05; **, p < 0.01; ***, p < 0.001; ****, p < 0.0001.

**Supplementary Figure 4**

**(A)** Schematic representation of the OVA/CpG vaccination model. A total of 100,000 CD45.1 OT-1 cells transduced with PGC-1α or SCR were transferred into CD45.2 naïve mice followed by peptide vaccination. **(B)** Percentages of transferred cells in blood at days 7, 13 and 30 postvaccination (% of CD8+) (pooled data from two experiments, 15 mice per group). **(C)** Expansion of transferred cells at 10 days post recall, expressed as fold change (6 mice per group). Data are representative of two independent experiments and are presented as the mean ± SD. *, p < 0.05; **, p < 0.01; ***, p < 0.001; ****, p < 0.0001.
