# Supplementary figures and images for: Enforced PGC-1α expression promotes CD8 T cell fitness, memory formation and antitumor immunity
Source: Cell Mol Immunol. 2020 Feb 13;18(7):1761–71. doi: 10.1038/s41423-020-0365-3 (PMC8245409; doi:10.1038/s41423-020-0365-3)

**A**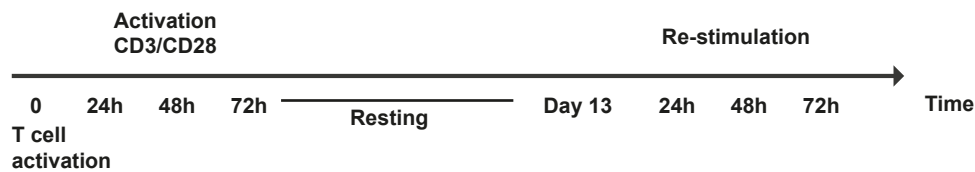**B**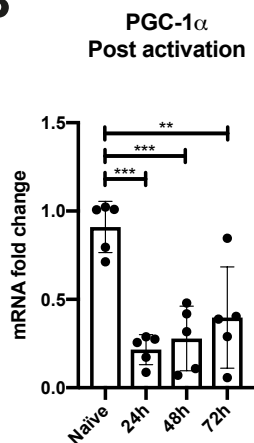**C**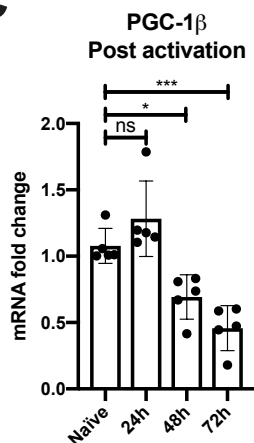**D**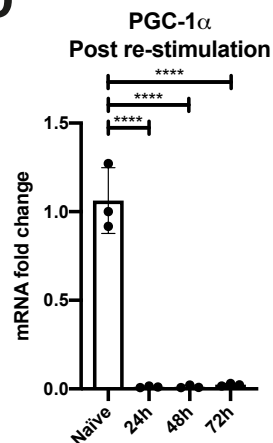**E**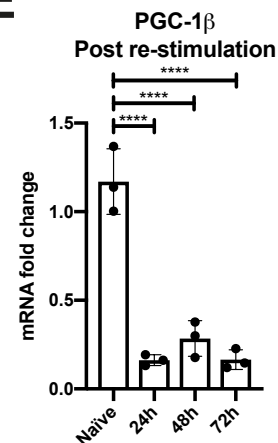**F**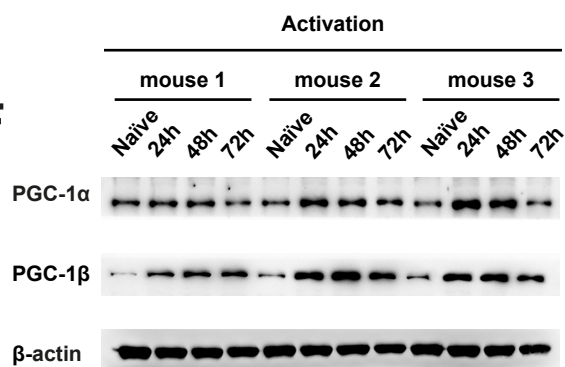**G**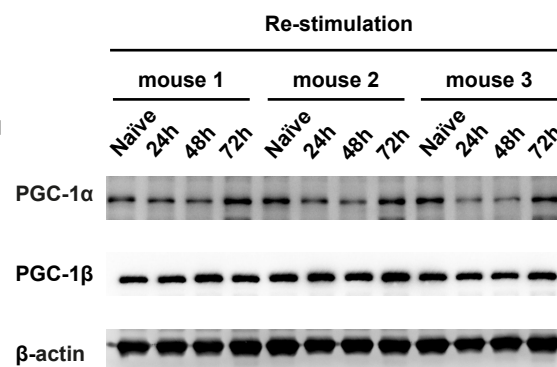**H**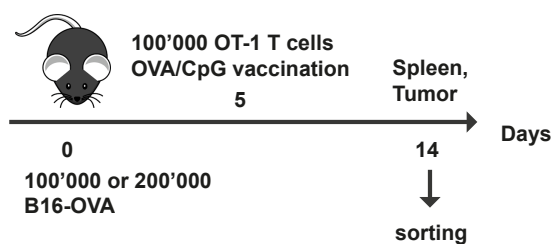**I**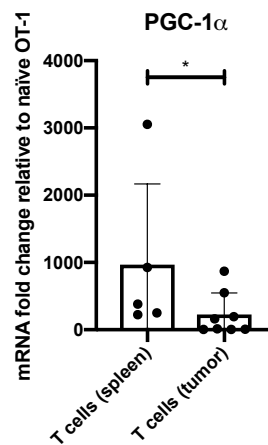**J**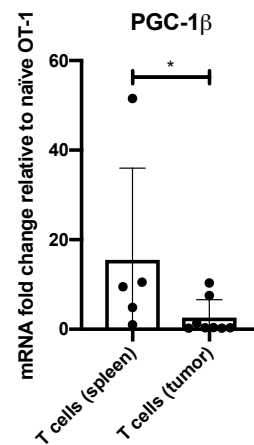**K**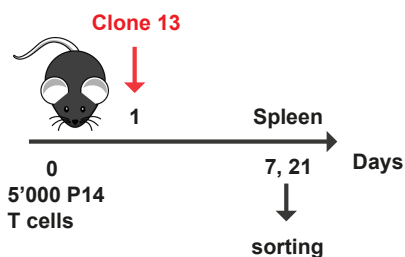**L**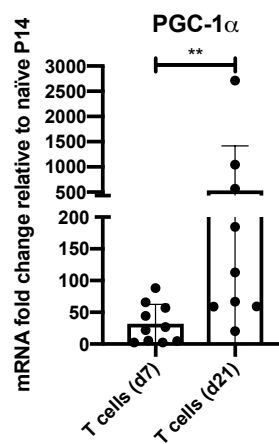**M**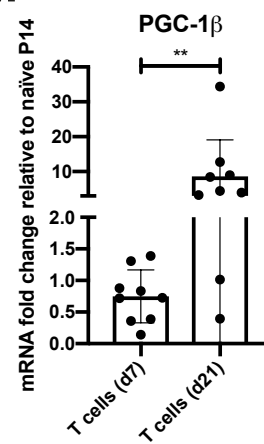

Supplement: Supplementary file 2 — Supplementary Figure 1 [file 41423_2020_365_MOESM2_ESM.pdf]

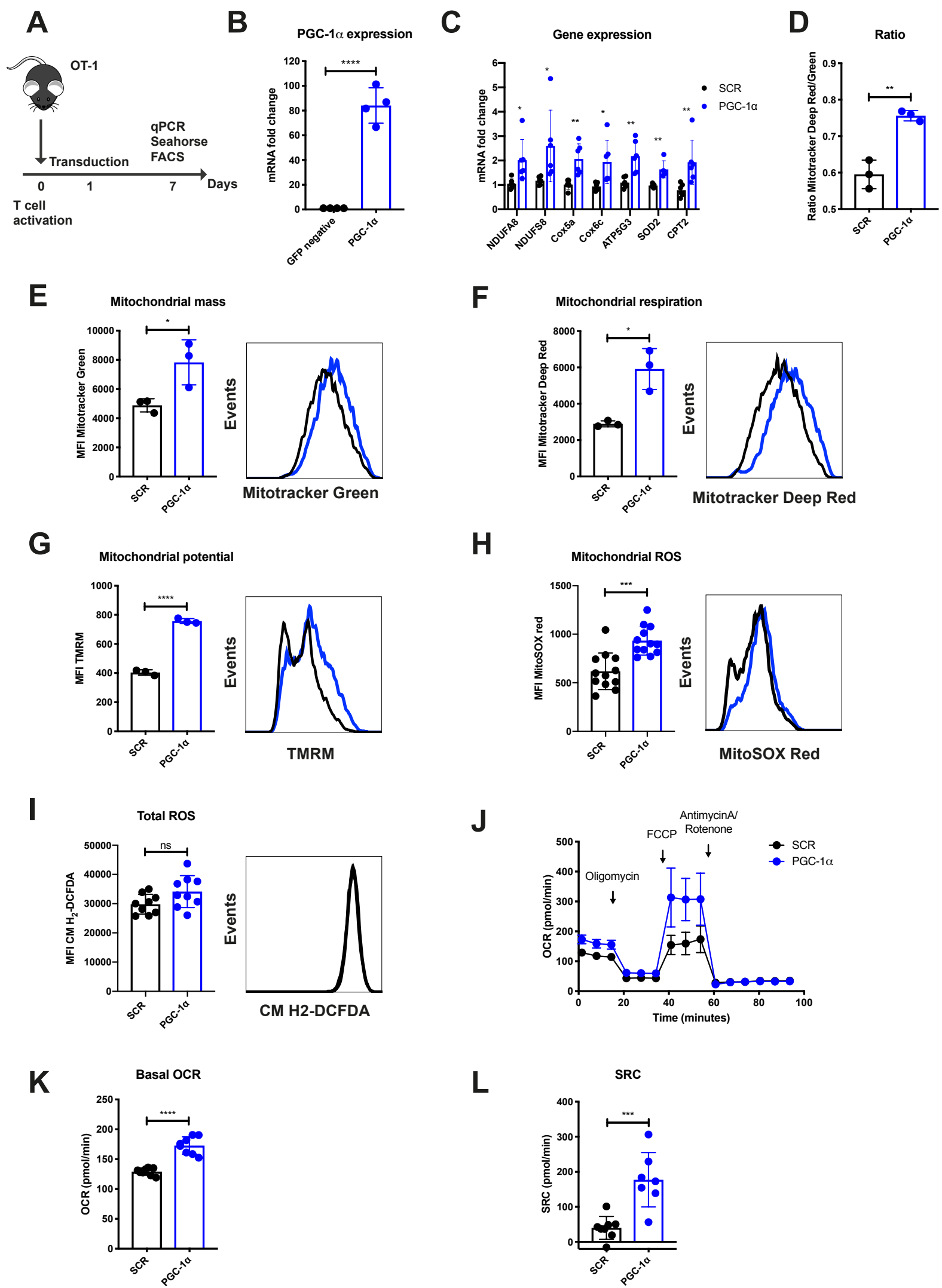

Supplement: Supplementary file 3 — Supplementary Figure 2 [file 41423_2020_365_MOESM3_ESM.pdf]
