# Supplementary material for: Enforced PGC-1α expression promotes CD8 T cell fitness, memory formation and antitumor immunity
Source: Cell Mol Immunol. 2020 Feb 13;18(7):1761–71. doi: 10.1038/s41423-020-0365-3 (PMC8245409; doi:10.1038/s41423-020-0365-3)
Supplement: Supplementary file 4 — Supplementary Figure 3 [file 41423_2020_365_MOESM4_ESM.pdf]

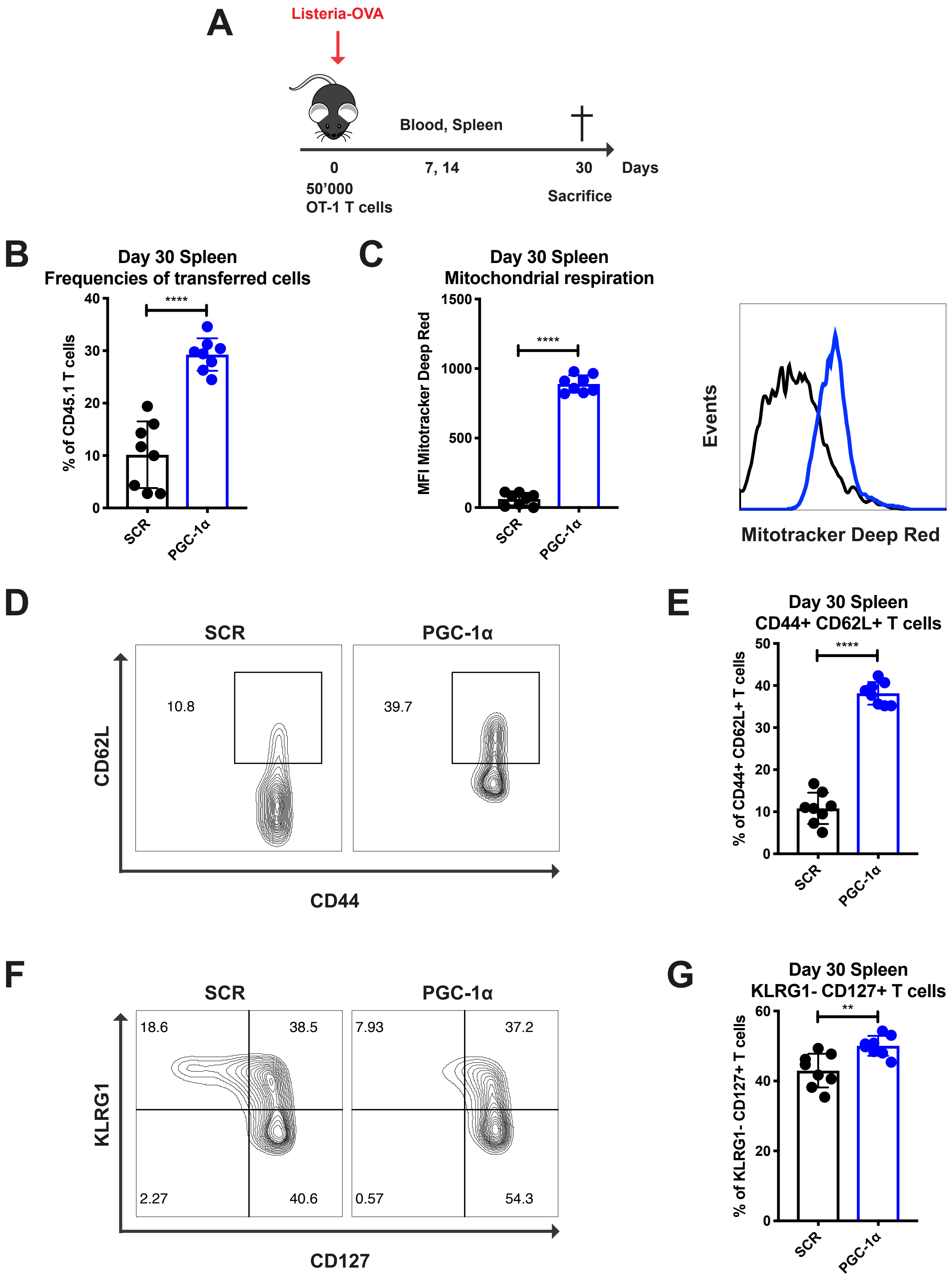
A
Listeria-OVA
Blood, Spleen
0
7, 14
30
Days
50'000
OT-1 T cells
Sacrifice
B
Day 30 Spleen
Frequencies of transferred cells
% of CD45.1 T cells
****
SCR
PGC-1α
C
Day 30 Spleen
Mitochondrial respiration
MFI Mitotracker Deep Red
****
SCR
PGC-1α
Events
Mitotracker Deep Red
D
SCR
PGC-1α
10.8
39.7
CD62L
CD44
E
Day 30 Spleen
CD44+ CD62L+ T cells
% of CD44+ CD62L+ T cells
****
SCR
PGC-1α
F
SCR
PGC-1α
18.6
38.5
2.27
40.6
7.93
37.2
0.57
54.3
KLRG1
CD127
G
Day 30 Spleen
KLRG1- CD127+ T cells
% of KLRG1- CD127+ T cells
**
SCR
PGC-1α
