# Supplementary material for: Enforced PGC-1α expression promotes CD8 T cell fitness, memory formation and antitumor immunity
Source: Cell Mol Immunol. 2020 Feb 13;18(7):1761–71. doi: 10.1038/s41423-020-0365-3 (PMC8245409; doi:10.1038/s41423-020-0365-3)
Supplement: Supplementary file 5 — Supplementary Figure 4 [file 41423_2020_365_MOESM5_ESM.pdf]

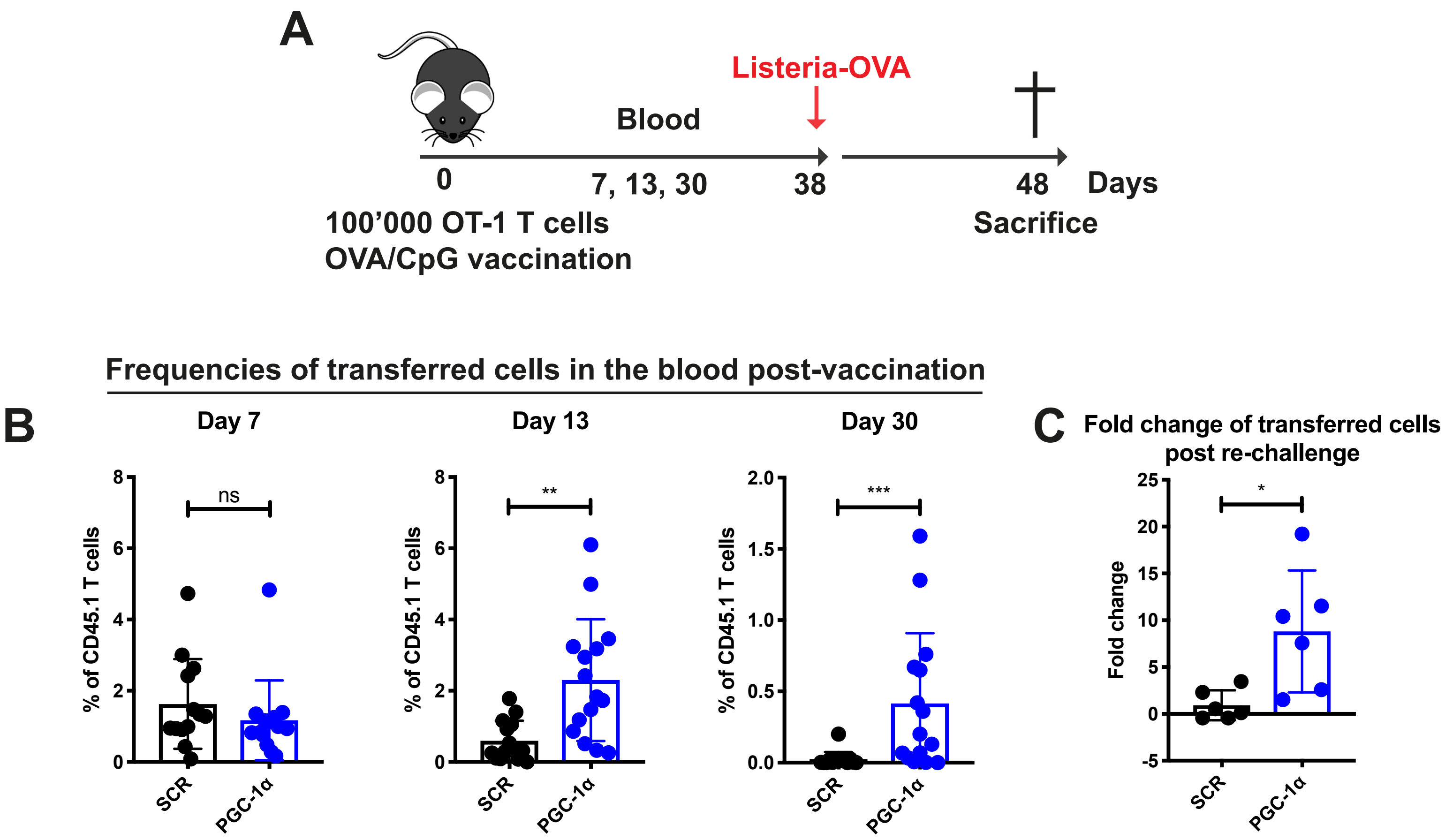
A
Listeria-OVA
Blood
0
7, 13, 30
38
48
Days
100'000 OT-1 T cells
OVA/CpG vaccination
Sacrifice
Frequencies of transferred cells in the blood post-vaccination
B
Day 7
Day 13
Day 30
% of CD45.1 T cells
ns
**
***
SCR
PGC-1α
C
Fold change of transferred cells post re-challenge
Fold change
*
